# B- and T cell receptor sequencing elucidates characteristics of lymphocyte depletion by ocrelizumab

**DOI:** 10.1016/j.isci.2025.113068

**Published:** 2025-07-07

**Authors:** Tilman Schneider-Hohendorf, Christian Wünsch, Andreas Schulte-Mecklenbeck, Lisa Revie, Catarina Raposo, Nicolas Strauli, Björn Tackenberg, Jan D. Lünemann, Catharina C. Gross, Luisa Klotz, Heinz Wiendl, Nicholas Schwab

**Affiliations:** 1 Department of Neurology, University Hospital Muenster, Muenster, Germany; 2 F. Hoffmann-La Roche Ltd, Basel, Switzerland; 3 Genentech, San Francisco, CA, USA; 4 Department of Neurology, Philipps-University, Marburg, Germany; 5 Department of Neurology and Neurophysiology, Medical Center, University of Freiburg, Freiburg, Germany

**Keywords:** Therapy, Immune response, Omics

## Abstract

Ocrelizumab therapy depletes circulating CD20^+^ lymphocytes. To characterize adaptive immune changes, we analyzed blood B- and T cell receptor repertoires of 35 multiple sclerosis (MS) patients before and alongside ocrelizumab therapy, compared to 11 healthy donors and 14 MS patients alongside natalizumab therapy, as well as cerebrospinal fluid and blood single-cell transcriptomics of 29 donors. Tissue-resident memory cells in cerebrospinal fluid revealed *MS4A1* gene expression and initial therapy-resistance. Depletion of CD20^+^ T cells was evident in highly expanded T-cell receptor beta chain and Vd2+ T-cell receptor delta chain clonotypes. Peripheral immunoglobulin heavy chain repertoires six months after initial depletion showed a bimodal patient distribution with either few B cells with high levels of somatic hypermutation, and comparatively high-sequence overlap with baseline samples, consistent with incomplete depletion of differentiated B cells or higher numbers of less differentiated B cells, indicating reconstitution from germline. Future studies will have to show how the identified phenotypes could be involved in MS pathology.

## Highlights

• *MS4A1* gene expression and initial therapy-resistance in CSF tissue-resident memory cells
• Ocrelizumab depletes highly expanded CD20dim alpha-beta and Vdelta2+ gamma-delta T cells
• After six months either few B cells with high somatic hypermutation and baseline overlap
• Or high numbers of less differentiated B cells, indicating reconstitution from the germline

## Graphical abstract

**Figure:**
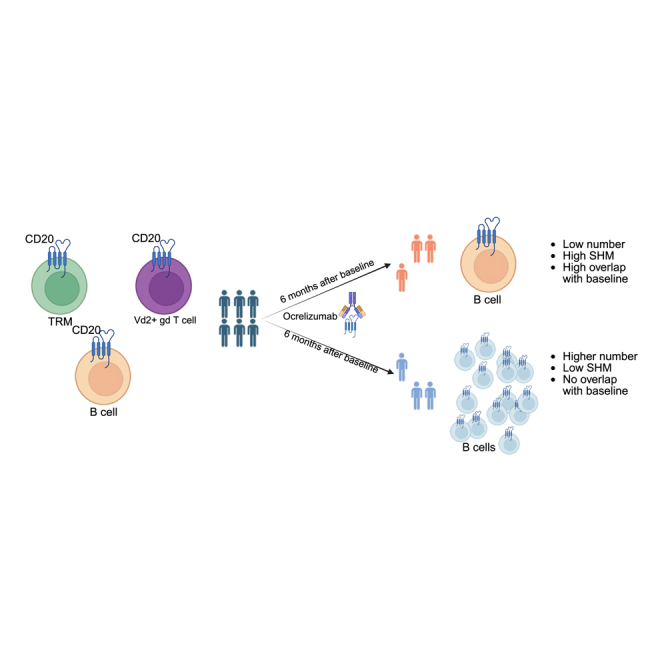

## Introduction

Multiple sclerosis (MS) is a chronic autoimmune disease of the central nervous system (CNS), characterized by formation of inflammatory lesions, mainly consisting of T cells and fewer B cells.^1^ However, the presence of immunoglobulin G (IgG) detectable as oligoclonal bands,^2^ as well as B- and plasma cells in cerebrospinal fluid (CSF) of MS patients,^3^ both used for diagnostic purposes, suggest that B cells contribute to MS pathology, as well. Despite the fact that B cell-depleting therapies such as ocrelizumab are effective treatments of MS, the exact role of B cells in MS pathophysiology has yet to be defined.^4^ The systemic humoral response is only slightly reduced by depletion of circulating B cells and not associated with the rapid clinical response.^5^ Intrathecal IgG antibodies may in part recognize ubiquitous intra-cellular auto-antigens^6^ but were also shown to be directed against the latent EBNA-1 epitope of the Epstein-Barr virus (EBV) with the potential for cross-reactivity to the CNS auto-antigen GlialCAM.^7^ Generally, the intrathecal IgG repertoire of MS patients was shown to be compartmentalized, putatively pathogenic,^8^ and capable of autonomously responding to local antigens,^9^ indicating a contribution of locally secreted antibodies by CNS-resident plasma cells to MS pathophysiology. Although it has been shown that B cells infiltrating the CSF of MS patients are reduced by ocrelizumab therapy,^10^^,^^11^^,^^12^ it is not clear to date whether the intrathecal IgG repertoire is significantly modulated by ocrelizumab therapy and is associated with clinical response.^13^^,^^14^

While treatment with ocrelizumab efficiently reduces MS relapse-associated worsening, some patients experience progression independent of relapse activity (PIRA).^15^^,^^16^ As ocrelizumab’s effect on non-circulating lymphocytes is limited, it is hypothesized that either incomplete depletion or reemergence of pathogenic lymphocytes could be the cause for PIRA alongside anti-CD20 therapy. Along the same lines, it was suggested that detectable B cells during ocrelizumab therapy are associated with more new/enlarging T2 lesions in RRMS patients, while detectable plasma cells could be associated with reductions in thalamic volume in PPMS patients.^17^ Therefore, it is currently evaluated whether higher dosing, changing of dosing intervals or personalized dosing according to B cell counts could improve patient care and efficacy alongside ocrelizumab therapy.^18^^,^^19^

Apart from antibody production, B cells can serve as antigen-presenting cells to T cells and secrete pro-inflammatory cytokines. Therefore, it is conceivable that therapeutic B cell depletion could also benefit MS patients by interfering with B cell/T cell interactions. Along the same line of argumentation, it has been suggested that B cell depletion diminishes the amount of putatively pathogenic, auto-proliferating CD4 T cells.^20^ Apart from its ubiquitous expression on B cells, CD20 is also known to be expressed to a lower extent (CD20dim) in a small subset of effector T cells, and these CD20dim T cells (∼5%–10% of T cells) are also depleted from circulation by anti-CD20 therapies.^21^^,^^22^ Interestingly, CD20dim T cells are enriched in CSF, as well as in lesions of MS patients with a tissue-resident memory (TRM) phenotype,^23^^,^^24^ and could be associated with MS disease severity,^11^^,^^25^^,^^26^^,^^27^ suggesting that part of B cell depletion efficacy might also be mediated by reduction of CD20dim T cells.

As anti-CD20 therapy in MS clearly affects peripheral B cells as well as T cells, we comprehensively analyzed the adaptive immune-receptor repertoire of both compartments before and six months after the start of ocrelizumab therapy by genomic DNA (gDNA) bulk sequencing in synopsis with flow cytometry data and CSF and peripheral blood single-cell RNA sequencing (scRNA-seq) data, to evaluate how ocrelizumab therapy modulates the B cell receptor (BCR) and T cell receptor (TCR) repertoire with special regard to therapy-resistant phenotypes.

## Results

### *MS4A1* is predominantly expressed- and persists in CSF T cells with a TRM phenotype

**Figure 1:**
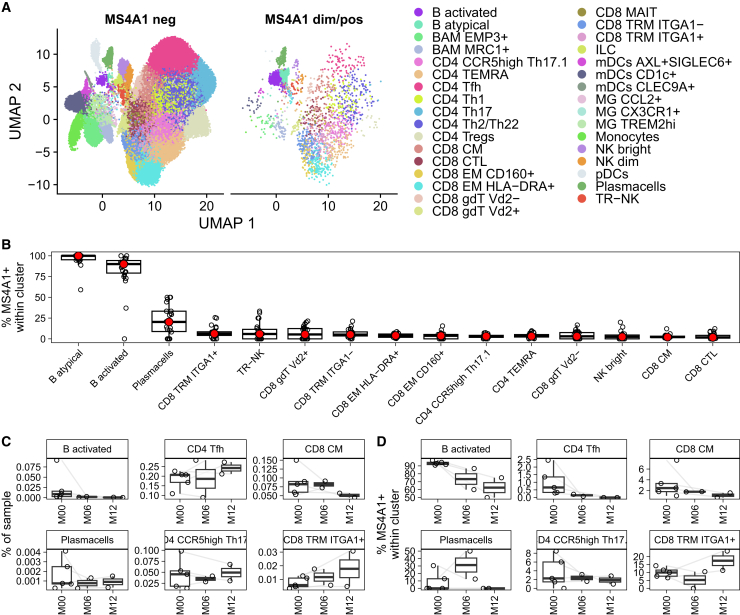
MS4A1 is predominantly expressed and persists in CSF T cells with a tissue-resident memory phenotype (A) UMAP plots of *MS4A1*-negative immune cells (MS4A1 neg, left panel) and *MS4A1*-positive CSF immune cells (MS4A1 dim/pos, right panel), annotated by *aCSF*^29^ and colored according to their immune cell subset annotations (legend). (B) Quantification of cell proportions expressing ≥1 *MS4A1* transcript counts within each immune cell subset per patient. Only subsets with ≥1% of *MS4A1-*expressing cells are shown. (C) Proportions of activated B cells (B activated), CD4^+^ Tfh (CD4 Tfh), CD8^+^ central-memory T cells (CD8 CM), plasma cells (Plasmacells), CD4^+^ CCR5high Th17.1 cells (CCR5high Th17.1) and CD8^+^ ITGA1+ TRM (CD8 TRM ITGA1+) in CSF before (M00), as well as six (M06) or twelve months (M12) alongside ocrelizumab therapy, quantified by *aCSF*.^29^ (D) Percentages of *MS4A1*+ cells within activated B cells (B activated), CD4^+^ Tfh (CD4 Tfh), CD8^+^ central-memory T cells (CD8 CM), plasma cells (Plasmacells), CD4^+^ CCR5high Th17.1 cells (CCR5high Th17.1) and CD8^+^ ITGA1+ TRM (CD8 TRM ITGA1+) in CSF before (M00), as well as six (M06) or twelve months (M12) alongside ocrelizumab therapy. Boxes indicate the 25% and 75% percentile and median, whiskers indicate 1.5x inter-quartile range.

Ocrelizumab depletes circulating CD20-expressing cells including CD20dim T cells.^22^^,^^28^ CD20dim T cells found in CNS lesions of MS patients were suggested to have a phenotype of TRM cells,^23^^,^^24^ indicating their involvement in CNS compartmentalized immunity due to chronic inflammation.^1^ We have previously characterized CD8^+^ and CD4^+^ subsets with a TRM phenotype in CSF and differentiated them from T cell subsets predominantly infiltrating the CSF from peripheral blood, such as B cell subsets, CD4^+^ follicular helper T cells (CD4^+^ Tfh) or CD8^+^ central-memory T cells.^29^ We then assessed which immune cell subsets express *MS4A1,* the gene encoding CD20, in previously published CSF scRNA-seq data of 24 MS patients from four independent datasets^30^^,^^31^^,^^32^^,^^33^ (Figure 1A). CSF immune cell subsets were divided in two groups: without any *MS4A1* expression versus with *MS4A1* counts >0. Quantification of subsets with *MS4A1-*positive cells showed that while CSF B cell subsets (activated and atypical B cells) presented with the highest proportion of *MS4A1*, 17% of CSF plasma cells also expressed *MS4A1*. Additionally, all subsets with a previously defined TRM phenotype (CD8 TRM and -EM, CD4 CCR5high Th17.1 and –TEMRA, tissue-resident NK cells [TR-NK]) as well as variable delta 2 chain (Vd2+) positive gamma-delta T cells (CD8 gdT Vd2+) and CD56 bright natural killer (NK) cell clusters contained ≥1% of *MS4A1*-expressing cells (Figure 1B). Interestingly, while almost all defined CD8^+^ subsets contained some *MS4A1*-expressing cells, only the CD4 subsets with TRM phenotype (CCR5high Th17.1 and TEMRA) contained >1% of *MS4A1* expressing cells.

We then compared previously defined CNS-resident immune cell subsets, such as plasma cells, CD4^+^ and CD8^+^ TRM (Plasmacells, CCR5high Th17.1 and CD8 TRM ITGA1+) to subsets of the respective lineage infiltrating the CSF from circulation, such as activated B cells, CD4^+^ Tfh, and CD8^+^ central-memory T cells (B activated, CD4 Tfh, CD8 CM). For this, we evaluated a published scRNA-seq dataset containing CSF and peripheral blood samples of RRMS patients before (five patients at M00) as well as either six months (two patients at M06) or twelve months (two patients at M12) alongside ocrelizumab treatment.^12^ Interestingly, besides B cells, none of the exemplary shown CSF immune cell subsets’ proportion was altered by ocrelizumab treatment (Figure 1C). However, the *MS4A1* expression was drastically reduced in CSF-infiltrating subsets, while *MS4A1* expression of CNS-resident immune cells was unaltered (Figure 1D). Accordingly, *MS4A1*-expressing CD4^+^ and CD8^+^ naive and memory T cells were depleted from circulation while *MS4A1*-negative T cells remained unaltered when using the same threshold of *MS4A1 >0/=0* (Figure S1A). Evaluation of the chosen threshold in an independent dataset, containing RNA and protein expression data from PBMCs of one healthy individual^34^ revealed sufficient congruence of RNA and protein expression with only 10.9% of *MS4A1*-classified negative being false-negative regarding CD20 protein expression, while 83.8% of *MS4A1*-classified positive cells being truly positive regarding CD20 protein expression in this donor (Figure S1B). Together, this confirms, that ocrelizumab-mediated depletion does not reach immune cell subsets with established CNS residency, at least in the first year of therapy, while *MS4A1*-expressing subsets infiltrating from the periphery, which could include TRM precursors, are reduced in CSF.

### Ocrelizumab-mediated B cell depletion elucidated by immunoglobulin heavy chain sequencing

**Figure 2:**
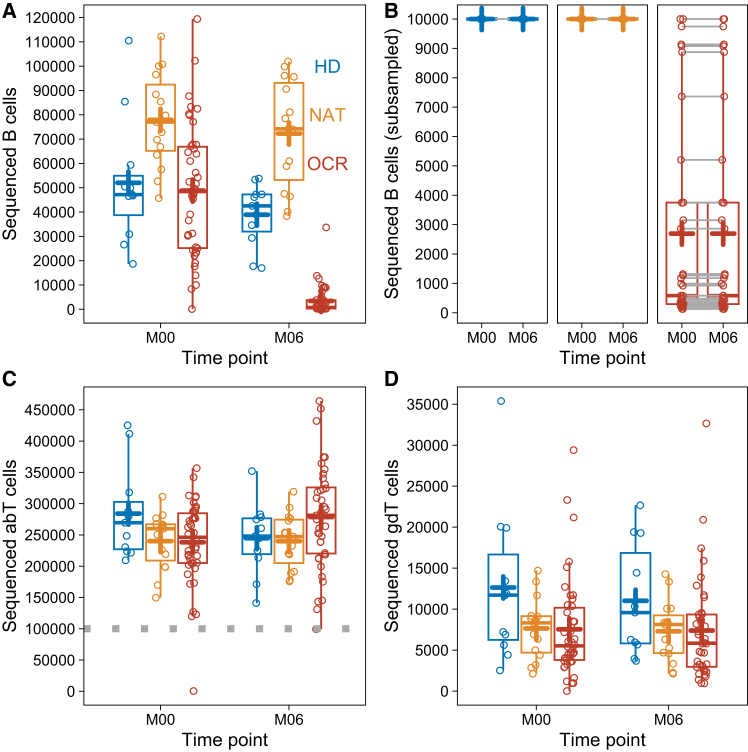
Study conception and sequencing downsampling strategies The plots depict IgH-expressing B cells, TRB-expressing alpha-beta T cells (abT cells) and TRD-expressing gamma-delta T cells (gdT cells), sequenced from gDNA of ten million cryopreserved PBMC in three cohorts: Healthy controls (HD) before the first (M00) and after the second SARS-CoV-2 mRNA vaccination (M06) (blue dots and boxes), MS patients during long-term natalizumab therapy (NAT) (M00 and M06 six months apart) (orange dots and boxes) and MS patients before (M00) and six months after the start of ocrelizumab therapy (OCR) (M06) (red dots and boxes). (A) Number of sequenced B cells by unique molecular identifiers. (B) Subsampling of HD and NAT to 10,000 sequenced cells and subsampling of M00 OCR to their respective M06 time points to account for depletion. (C) Number of sequenced alpha-beta T cells by unique molecular identifiers. Subsampling to 100,000 cells for consecutive analyses is indicated by gray dots. (D) Number of sequenced gamma-delta T cells by unique molecular identifiers. Boxes indicate the 25% and 75% percentile and median, whiskers indicate 1.5x inter-quartile range, + indicates the mean.

**Figure 3:**
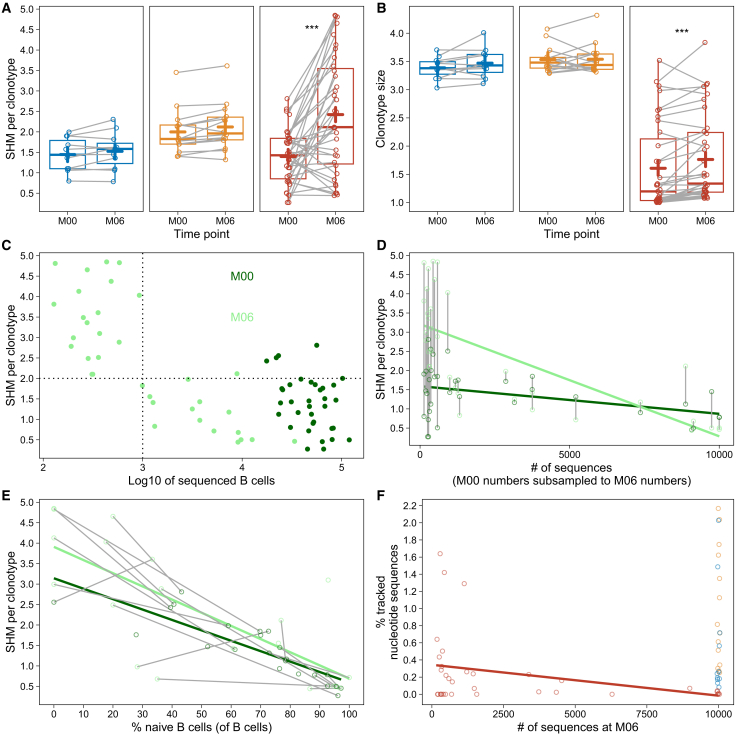
The BCR repertoire architecture six months after depletion depends on the level of B cell reconstitution The plots depict IgH clonotype metrics, where a IgH clonotype is defined by IgH sequences grouped into one phylogenetic tree. B cells were sequenced from gDNA of ten million cryopreserved PBMC in three cohorts: Healthy controls (HD) before the first (M00) and after the second SARS-CoV-2 mRNA vaccination (M06) (blue dots and boxes), MS patients during long-term natalizumab therapy (NAT) (M00 and M06 six months apart) (orange dots and boxes), and MS patients before (M00) and six months after the start of ocrelizumab therapy (OCR) (M06) (red dots and boxes). (A) Mean somatic hypermutations (SHM) per clonotype. (B) Mean clonotype size defined by clonotype member sequence count. (C) Mean SHM per clonotype (*y* axis) correlated to the numbers of IgH sequences (*x* axis) from Figure 2A. (D) Mean SHM per clonotype (*y* axis) correlated to the numbers of IgH sequences at M06 (*x* axis) from Figure 2B, where M00 is subsampled to M06; Dark green dots indicate M00 SHM levels, light green dots indicate M06 SHM levels, gray lines connect patients’ M00 and M06 values, the dark green line depicts linear regression of M00 SHM levels and the light green line depicts linear regression of M06 SHM levels, both in relation to sequenced B cells at M06. (E) Mean SHM per clonotype (*y* axis) correlated to proportions of naive B cells of total B cells at M06 (*x* axis), measured by flow cytometry; Dark green dots indicate SHM levels and naive B cell proportions at M00, light green dots indicate SHM levels and naive B cell proportions at M06, gray lines connect patients’ M00 and M06 values, the dark green line depicts linear regression of M00 values and the light green line depicts linear regression of M06 values. (F) Sample overlap, defined as the proportion of the M06 sample that contained nucleotide sequences already known from a fixed number of 10^4^ IgH sequences from M00 (*y* axis), correlated to the number of IgH sequences of M06 (*x* axis) from Figure 2A, the red line indicates the linear regression. ∗∗ indicates a *p* value of <0.01, ∗∗∗ indicates a *p* value of <0.001. Boxes indicate the 25% and 75% percentile and median, whiskers indicate 1.5x inter-quartile range, + indicates the mean.

Bulk sequencing from PBMCs was then used to analyze how lymphocyte depletion by ocrelizumab influenced the architecture of the BCR and TCR repertoire. Three groups of individuals were analyzed: 35 MS patients before (M00) and six months (M06) after the first ocrelizumab infusion (OCR); 11 healthy donors with two time points (before and after the second SARS-CoV-2 vaccination with an mRNA vaccine, HD); 14 MS patients during two time points (6 months apart, NAT) of long-term therapy with natalizumab. The HD group was chosen to reflect a healthy repertoire with antigen exposure over time, the NAT group was chosen as comparator alongside a stable therapy, which is known to sequester B cells over T cells in peripheral blood.^35^ The initial ocrelizumab treatment cycle reduced the amount of sequenced B cells in a sample from 53,670 to 4,408 sequenced cells (92% reduction; p = 4 × 10^−10^), while NAT samples expectedly presented with higher numbers of sequenced B cells compared to HD (77,601 sequenced cells; *p* = 0.002) (Figure 2A). To allow a detailed analysis of the immunoglobulin heavy chain (IgH) BCR repertoire, OCR M00 samples were downsampled to their respective M06 follow-up sample, or to 10,000 cells if available at M06. The HD and NAT samples were also downsampled to 10,000 cells (Figure 2B). For TCR analysis, the variable beta chain (TRB) of alpha-beta T cells and the delta chain (TRD) of gamma-delta T cells were sequenced. Amounts of sequenced TRB T cells between donors were compensated by downsampling to 100,000 T cells per sample (Figure 2C), sequenced TRD T cells are depicted in Figure 3D.

### The BCR repertoire architecture six months after depletion depends on the level of B cell reconstitution

Consistent with reports of higher memory B cell proportions in peripheral blood,^36^^,^^37^ clonotypes of the NAT group, defined by IgH sequences grouped into one phylogenetic tree, showed higher levels of somatic hypermutation (SHM) compared to HD (*p* = 0.013). Previous reports assessing the B cell repertoire after initial depletion by ocrelizumab using flow cytometry mostly detected transitional B cells in circulation, indicating reconstitution of the BCR repertoire from the bone marrow.^22^^,^^38^ However, after B cell depletion IgH sequencing analysis detected an overall increase of SHM per clonotype in the majority of the OCR group (Figure 3A). Additionally, these clonotypes with high levels of SHM were not isolated cells but larger than clonotypes subsampled from baseline (Figure 3B). The distribution of clonotype size measured by clonality was also shifted toward more perturbation after ocrelizumab therapy (Figure S2). Together these measurements suggested that the BCR architecture after six months of ocrelizumab therapy reflected a more mature B cell phenotype in the majority of patients. However, as there was a bimodal distribution of patients after B cell depletion, the amount of SHM was correlated to the levels of peripheral B cells (proxied by number of sequenced B cells in 1 × 10^7^ PBMC) (Figure 3C). This is especially interesting, as large studies have used the threshold of flow cytometry-derived 10 B cells per μL to deem the level “detectable”.^17^^,^^18^ After correlating measured B cell counts from fresh blood with the number of sequenced B cells in a sample (Figure S3A), it became evident, that 10 B cells per μL would correspond to roughly 5,000 sequenced B cells in a sample. To divide the bi-modal distribution, we would have to set the threshold at 1,000 sequenced B cells in a sample, which would be roughly 1 B cell per μL, to consider a patient to have a reconstructed repertoire. To make sure that this finding was not artificially created by the low number of analyzed B cells, we correlated the number of analyzed B cells and level of SHM and could show that the negative correlation was only visible at time point M06 and not at baseline, even though the same number of cells were analyzed (Figure 3D). As this suggested that in patients with higher number of peripheral B cells six months after depletion the repertoire consisted of mainly naive B cells, this was correlated with flow cytometry measurements, which showed that the level of SHM inversely reflected the proportion of naive B cells (Figure 3E). Interestingly, the number of trackable nucleotide sequences also inversely correlated with the number of sequenced B cells, indicating that high-SHM clonotypes rather consist of sequences from differentiated B cells or plasma cells, which could already be detected at baseline. Low-SHM clonotypes, which were associated with generally higher levels of B cells after depletion, could not be traced back to baseline, indicating reconstitution from bone marrow (Figure 3F). This bimodal distribution of B cells could be reproduced in an independent dataset, containing scRNA-seq from blood of 18 MS patients six months after the initial ocrelizumab cycle,^12^ confirming that patients either show low amounts of putatively non-depleted plasma blasts or higher counts of putatively naive B cells (Figure S3B). Together, the data indicate that patients with hardly any detectable B cells after depletion only present with putatively non-depleted memory B cells or plasma cells in peripheral blood, whereas comparatively higher number of B cells are mostly naive and very close to the germline. In line with a previous study, the heavy chain variable gene distribution of the B cells remaining after depletion did not differ significantly from the baseline in any group^39^ (Figure S4).

### Ocrelizumab treatment depletes highly expanded alpha-beta and Vd2+ gamma-delta T cells

**Figure 4:**
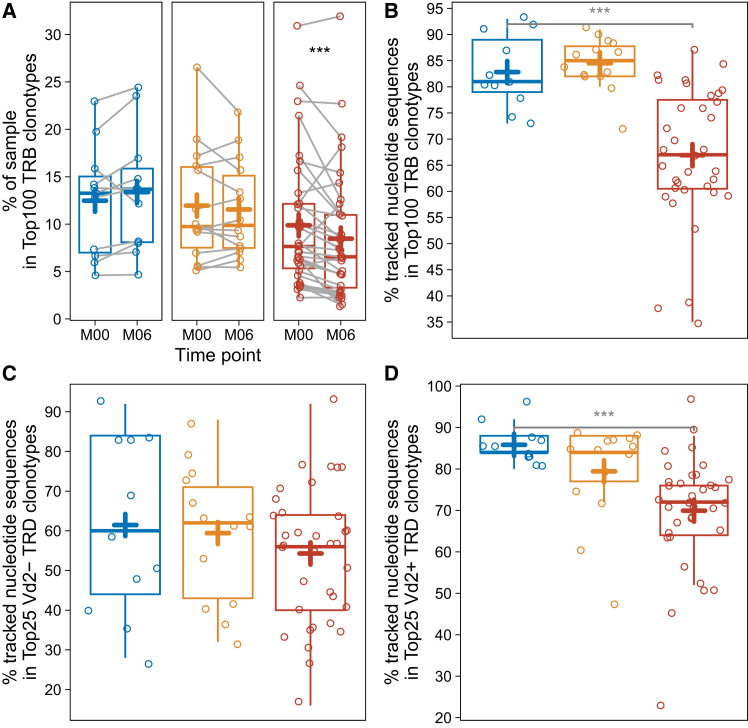
Ocrelizumab treatment depletes highly expanded alpha-beta T cells and Vd2+ gamma-delta T cells The plots depict metrics of the TRB and TRD repertoires, where a clonotype is defined by identical nucleotide sequence: T cells were sequenced for TRB and TRD loci from gDNA of ten million cryopreserved PBMC in three cohorts: healthy controls (HD) before the first (M00) and after the second SARS-CoV-2 mRNA vaccination (M06) (blue dots and boxes), MS patients during long-term natalizumab therapy (NAT) (M00 and M06 six months apart) (orange dots and boxes) and MS patients before (M00) and six months after the start of ocrelizumab therapy (OCR) (M06) (red dots and boxes). (A) Proportion of TRB sample occupied by the top 100 clonotypes. (B) Proportions of top 100 TRB clonotypes at M06 already detected in the top 100 TRB clonotypes of M00. (C) Proportions of top 25 TRD Vd2-clonotypes at M06 already detected in the top 25 TRD Vd2-clonotypes of M00. (D) Proportions of top 25 TRD Vd2+ clonotypes at M06 already detected in the top 25 TRD Vd2+ clonotypes of M00. ∗∗ indicates a *p* value of <0.01, ∗∗∗ indicates a *p* value of <0.001. Boxes indicate the 25% and 75% percentile and median, whiskers indicate 1.5x inter-quartile range, + indicates the mean.

It was previously shown that ocrelizumab therapy also depletes circulating, putatively pathogenic CD20dim T cells, which are differentiated, predominantly CD8^+^ memory T cells,^22^^,^^40^ and were suggested to express both alpha-beta and gamma-delta TCRs.^41^ Secondly, a depletion-induced lack of B cell antigen presentation to T cells could alter the TCR repertoire. Therefore, the TRB and TRD repertoire architecture was assessed for putative modulation by ocrelizumab therapy. Although the general architecture in terms of clonality was unaltered for TRB and TRD (Figures S5A and S5B), in-depth analysis of clonal distribution revealed reduction of the top 100 TRB T cell clonotype proportions (Figure 4A), which would be in line with depletion of expanded TRB CD20dim T cells. Additionally, nucleotide sequence overlap of the top 100 TRB T cell clones was reduced by ocrelizumab, indicating depletion of some top expanded TRB clonotypes. (Figure 4B). This could be confirmed by correlating flow cytometry-derived CD19^−^ CD20^+^ lymphocyte percentages with TRB top expanded clonal proportions (Figure S5C). Further validation was generated by an independent scRNA-seq dataset including TCR data of paired blood and CSF samples from five MS patients and six healthy controls^42^: Expanded TRB clonotypes showed higher proportion of *MS4A1* expressing cells both in blood and CSF (Figure S5D), and *MS4A1*-expressing, expanded clonotypes showed sequence overlap from blood to CSF (Figure S5E). This indicates that top expanded clonotypes are enriched for CD20dim T cells and that CD20dim clonotypes expanded in blood infiltrate the CNS and can, therefore, be detected in CSF.

As we had shown that also Vd2+ gamma-delta T cells express *MS4A1*, nucleotide sequence tracking was performed separately for Vd2-and Vd2+ gamma-delta T cells. In line with their *MS4A1* expression, only the nucleotide sequence overlap of Vd2+ gamma-delta T cells was reduced within the top 25 expanded clones, indicating specific depletion by ocrelizumab (Figures 4C and 4D).

## Discussion

Ocrelizumab efficiently depletes B cells from circulation, while non-circulating B cells, as well as CD20^−^ B cells, i.e., part of plasma cells, are spared from depletion.^43^ Reappearing B cells, assessed before the next depletion cycle, were reported to mainly consist of transitional or naive B cells,^17^^,^^22^^,^^44^^,^^45^ indicating reconstitution from bone marrow. However, also small amounts of plasma cells and memory B cells, particularly CD11c+ B cells, could be detected.^17^^,^^22^^,^^46^^,^^47^^,^^48^ The presented IgH repertoire sequencing can confirm both observations but puts them in context of fresh blood B cell counts: samples with low number of circulating B cells six months after the first depletion cycle contained mostly IgH sequences with high numbers of SHM. These clonotypes were already present before the start of ocrelizumab therapy, are likely highly differentiated, non-depleted memory B cells and/or plasma cells^46^^,^^48^ but do not share common germline ancestries between patients.^39^ However, in samples with higher B cell counts, those highly differentiated B cells were overshadowed by the number of naive B cells with low/no SHM IgH sequences, which were not detectable at baseline, indicating fast B cell reconstitution from bone marrow in these patients. Retrospectively, according to the current flow cytometry-based cutoff of <10 B cells per μL in our real-world cohort, six of 26 patients exhibited increased B cell reconstitution while IgH sequencing would identify twelve of 26 patients with increased B cell reconstitution. This might have implications for personalized dosing approaches,^19^ since in several large ocrelizumab trials high lesion load/MRI activity correlated with detectable B cells in RRMS patients alongside ocrelizumab therapy.^17^ For the level of detection, most studies have used 10 B cells per μL. Our data suggest that, although not applicable in clinical routine, biologically, this threshold could be lowered to 1 B cell per μL, as this is where the bi-modal distribution of patients was divided. Of note, highly differentiated B cells were shown to decline with consecutive cycles of ocrelizumab-mediated B cell depletion,^48^ indicating that initially therapy-resistant B cells recirculate or re-express CD20.

Regarding T cells, we could not find evidence that B cell depletion profoundly alters the TCR repertoire by lack of B cell-mediated antigen presentation, confirming a prior study.^46^ However, it was previously shown that apart from B cells, also CD20dim T cells (5%–10% of T cells) are depleted by ocrelizumab therapy, predominately within the CD8 lineage.^22^ Within the TCR repertoire, we found evidence that ocrelizumab reduces and partly depletes highly expanded TRB and Vd2+ TRD clonotypes, which could be traced back to CD20dim T cells using flow cytometry parameters. Clonal expansions of CD20dim T cells in blood and CSF could be confirmed in an independent dataset.^42^ CD20dim T cells could be implicated in several autoimmune diseases,^40^ were shown to be memory cells with a pro-inflammatory, CNS-homing phenotype in MS patients,^21^^,^^22^^,^^26^ and are enriched in RRMS and PPMS CSF.^25^^,^^27^ CD20dim T cells were also detected in healthy donor CNS and white matter lesions of MS patients, with a presumed phenotype of TRM.^23^^,^^24^ However, whether TRM only contribute to pathogenic processes with regard to chronic CNS diseases still needs to be determined.^49^ Our transcriptomic analysis revealed that also in CSF a high percentage of CD8^+^ TRM expresses CD20. CD8^+^ TRM were shown to populate the CSF even when peripheral lymphocyte infiltration is halted.^29^ Similarly, and in contrast to CD4^+^ CD20dim T cells, CD8^+^ CD20dim T cells in MS CSF are not depleted by dimethyl fumarate treatment,^27^ which selectively depletes activated memory lymphocytes from circulation.^50^ Accordingly, treatment with ocrelizumab was shown to only diminish CD4^+^ CD20dim cells in CSF, while CD8^+^ CD20dim TRM are conserved, although both populations are efficiently depleted from circulation.^11^^,^^22^ Our transcriptomic analysis now also showed that specifically cells infiltrating the CSF, such as B cells, CD4^+^ Tfh, or CD8^+^ central-memory T cells did not express CD20-encoding *MS4A1* alongside ocrelizumab therapy, while its expression was unaltered in CSF lymphocytes with a TRM phenotype, at least in the first year of therapy. Together, this suggests that CSF-populating TRM, including CD8^+^ CD20^+^ TRM, are not regularly replenished from circulation, but have the ability to self-maintain intrathecally. Increased presence of CD20dim T cells in CSF was found to correlate with disease progression in MS,^25^^,^^27^ indicating establishment of compartmentalized CNS immunity over the course of the disease.

Apart from CD8^+^ TRM, we also detected *MS4A1* expression and depletion specifically in Vd2+ gamma-delta T cells, while Vd2-gamma-delta T cells appeared unaltered. In line with this observation, it was suggested before that CD20 is expressed in higher proportions in gamma-delta T cells compared to alpha-beta T cells,^41^ and previous studies indicated depletion of gamma-delta T cells from periphery,^22^ as well as from CSF^12^ alongside treatment with ocrelizumab. Lastly, we detected *MS4A1* expression also in tissue-resident and CD56 bright NK cells of MS CSF, and CD20 expression and potential modulation of NK subsets by ocrelizumab therapy was observed before.^51^^,^^52^^,^^53^

Taken together, sequencing of the BCR and TCR repertoire alongside ocrelizumab therapy strengthened the idea of near total depletion of circulating B cells. This depletion did not fundamentally alter the TCR repertoire suggesting that a potential lack of B cell mediated antigen presentation to T cells does not interfere with T cell homeostasis, at least in the short period of observation. We could show that CD20 low-expressing lymphocytes including CD8^+^ alpha-beta T cells and Vd2+ gamma-delta T cells are depleted from circulation as well. We found that these CD20dim T cells in the CSF of MS patients predominantly show a phenotype of tissue-resident memory cells and are not depleted by ocrelizumab. Detectable B cell levels before the next infusion in half of the patients were not due to insufficient depletion but consisted of previously undetected B cells newly generated from the germline and potentially the bone marrow. The other half of the patients only presented with very low levels of highly differentiated B cells. These clonotypes could be traced back to baseline, are therefore highly likely non-depleted, putatively non-circulating cells. To prevent the establishment of pathological CNS-resident immunity in MS could be one angle to reduce future risk of progression.

### Limitations of the study

For this study, only the baseline and the six-month time point after the start of ocrelizumab therapy were investigated by newly generated, immune receptor bulk sequencing data. Additionally, flow cytometry data were restricted to immune cell lineage markers and a small set of B cell subset-defining markers. Therefore, immune receptor sequencing phenotypes could not be fully resolved by flow cytometry-defined immune cell subsets. Lastly, the MS patient cohort size was not sufficient to robustly correlate the identified sequencing phenotypes with clinical parameters.

## Resource availability

### Lead contact

Further information and requests for resources and data should be directed to and will be fulfilled by the lead contact, Dr. Nicholas Schwab (nicholas.schwab@ukmuenster.de).

### Materials availability

This study did not generate new unique reagents.

### Data and code availability

Sequenced immune receptor repertoire datasets have been deposited to Mendeley Data and are publicly available. Accession numbers are listed in the key resources table. Previously published scRNA-seq datasets with accession numbers are listed in the key resources table.

This paper does not report original code. Previously published code (*aCSF*) was used to analyze CSF scRNA-seq data,^29^ also referenced in the key resources table. Any additional information required to reanalyze the data reported in this paper is available from the lead contact upon request.

## Acknowledgments

We would like to thank Eva Maria Schumann, Barbara Meyring and Petra Kotte for their help with biobanking and sample handling. We would like to thank Marianne Riepenhausen, Florian Rubelt, Hamid Mirebrahim, and Hosseinali Asgharian for their help with sequencing data preprocessing. This study was funded by the Deutsche Forschungsgemeinschaft National Research Data Infrastructure (NFDI) for Immuno (grant number 501875662) and F. Hoffmann-La Roche Ltd., Basel, Switzerland as part of Integrative Neuroscience Collaborations Network. Graphical abstract was created in BioRender https://BioRender.com/f5ih8cs.

## Author contributions

T.S.-H., conceptualization, methodology, formal analysis, investigation, visualization, and writing; C.W., resources, data curation, methodology, and writing; A.S.-M., resources, formal analysis, and writing; L.S., resources, data curation, and writing; C.R., conceptualization, investigation, formal analysis, supervision, and writing; N. Strauli, methodology, formal analysis, investigation, and writing; B.T., conceptualization, supervision, and writing; J.D.L., resources, investigation, supervision, and writing; C.C.G., resources, formal analysis, supervision, and writing; L.K., conceptualization, resources, investigation, supervision, and writing; H.W., funding acquisition, conceptualization, resources, supervision, and writing; N. Schwab, funding acquisition, conceptualization, methodology, investigation, formal analysis, visualization, supervision, and writing. All authors agreed to submit the manuscript, read and approved the final draft, and take full responsibility of its content, including the accuracy of the data and its statistical analysis. T.S.-H. and N.S. performed, oversaw, and replicated statistical analyses, had unrestricted access to all data, and prepared the first draft of the manuscript, reviewed and edited it.

## Declaration of interests

T.S.-H. received a travel grant from F. Hoffmann-La Roche Ltd associated with the study, and research support from Novartis outside of the submitted work. C.R. is an employee of F. Hoffmann-La Roche Ltd. N. Strauli is an employee of Genentech. B.T. is an employee of F. Hoffmann-La Roche Ltd. J.D.L. received speaker fees, research support, travel support, and/or served on advisory boards by Abbvie, Alexion, Adivo, Amgen, Argenx, Biogen, CSL Behring, Janssen-Cilag, Merck, Moderna, Novartis, Roche, Sanofi, Takeda and UCB Pharma. C.C.G. received grants from Biogen, F. Hoffmann-La Roche Ltd and Novartis Pharma; and consultancy fees from MyLan and DIU Dresden International University GmbH. L.K. received fees from Alexion, Bayer, Biogen, Celgene, Sanofi, Horizon, Grifols, Merck-Serono, Novartis, F. Hoffmann-La Roche Ltd, Santhera and Teva; and research grants from Biogen, Immunic AG, Novartis and Merck-Serono. H.W. received consultancy fees for Abbvie, Alexion, Argenx, Biogen, Bristol Myers Squibb/Celgene, EMD Serono, F. Hoffmann-La Roche Ltd, Fondazione Cariplo, Genzyme, Gossamer Bio, Idorsia, Immunic, Immunovant, Janssen, Lundbeck, Merck, Neurodiem, NexGen, Novartis, PSI CRO, Roche Pharma AG, Sanofi, Swiss Multiple Sclerosis Society, Teva, UCB Biopharma, WebMD Global and Worldwide Clinical Trials; and research grants from Deutsche Myasthenie Gesellschaft e.V., Alexion, Amicus Therapeutics Inc., Argenx, Biogen, CSL Behring, F. Hoffmann-La Roche Ltd, Genzyme, Merck, Novartis Pharma, Roche Pharma AG and UCB Biopharma outside of the submitted work. N. Schwab received grants from Biogen outside the submitted work and from F. Hoffmann-La Roche Ltd. associated with the study.

## STAR★Methods

### Key resources table

**Table:** 

REAGENT or RESOURCE	SOURCE	IDENTIFIER
**Biological samples**		

MS patient and HD PBMC samples	University Hospital Muenster	NA

**Deposited data**		

MS patient blood TRB and IgH bulk sequencing data	This publication	Mendeley Data: https://doi.org/10.17632/d7p99vzcb6.1
RRMS patient blood and CSF scRNAseq data	Wei et al.^12^	dbGaP: phs003938.v1.p1
RRMS patient CSF scRNAseq data	Ramesh et al.^30^	GEO: GSE133028
RRMS patient CSF scRNAseq data	Esaulova et al.^31^	Synapse.org: syn21904732
RRMS patient CSF scRNAseq data	Heming et al.^32^	GEO: GSE163005
RRMS patient CSF scRNAseq data	Schafflick et al.^33^	GEO: GSE138266
RRMS patient and HD CSF scRNAseq data	Pappalardo et al.^52^	dbGaP: phs002222.v2.p1
HD scRNAseq and CITEseq data	10x Genomics^34^	80k_Human_PBMCs

**Software and algorithms**		

aCSF	Ostkamp et al.^29^	schwab-lab/annotateCSF
R v4.3.0.	R Core Team	r-project.org
Immcantation framework	Vander Heiden et al.^54^	immcantation
changeo IgBLAST function v4.4.0	Gupta et al.^55^	immcantation/changeo
SCOPer v1.3.0	Nouri et al.^56^	immcantation/scoper
Dowser v2.2.0	Hoehn et al.^57^	immcantation/dowser
SHazaM v1.2.0	Gupta et al.^55^	immcantation/shazam

### Experimental model and study participant details

#### Study cohorts and controls

The cohort consisted of 35 MS patients (25 RRMS, 10 PPMS, 23 female, 12 male) with a mean age of 40.46 ± 1.92, locally recruited for an investigator-initiated trial before the start of treatment with ocrelizumab (baseline, M00), and six months alongside treatment with ocrelizumab (follow-up, M06). As controls, 11 healthy donors (mean age 40 ± 4.27, 5 female, 6 male) before (M00) and up to six months later, after the second anti-SARS-CoV-2 mRNA vaccination (M06) and 14 RRMS patients alongside long-term treatment with natalizumab (mean age 39.01 ± 1.84, 8 female, 6 male), six months apart (M00, M06) were analyzed. Race, ancestry, ethnicity and socioeconomic status of study donors were not available due to data privacy.

#### Ethics approval

All patients gave written informed consent to sample collection and data analysis. The study was approved by the local ethics committee in Muenster, Germany (Ethics Committee of the Board of Physicians of the Region Westfalen-Lippe and of the University of Muenster, reference number 2015-522-f-S) and the study was conducted in accordance with the Declaration of Helsinki.

### Method details

#### Biomaterial processing

Peripheral blood mononuclear cells (PBMC) were isolated from peripheral blood by density centrifugation with subsequent cryopreservation of 10 million PBMC per sample as described previously.^58^ Genomic DNA (gDNA) was isolated from thawed PBMC using the DNeasy blood and tissue kit (Qiagen) according to the manufacturer’s instructions.

#### Flow cytometry

Fresh blood 10-color flow cytometry was performed as described previously^3^ with two different antibody panels: Immune cell lineages (anti-CD14, anti-CD138, anti-HLA-DR, anti-CD3, anti-CD56, anti-CD4, anti-CD19, anti-CD16, anti-CD4 and anti-CD45) and B-cell subpopulations (anti-IgD, anti-CD138, anti-CD20, anti-CD38, anti-CD27, anti-CD23, anti-CD19, anti-PD-1,-CD5 and anti-CD45. Analysis of flow cytometry data was performed with Kaluza V2.1.

#### Immune receptor repertoire sequencing and analysis

Sequencing was performed on PBMC gDNA (ImmunoPETE assay, Roche) as described previously.^59^ BCR analysis was performed using R 4.3.0., the Immcantation framework and its Docker container.^54^^,^^55^ Phylogenetic trees of IgH sequences were assembled with a spectral clustering-based method.^56^ In short, productive BCR nucleotide sequences were subsampled to the specified amount, annotated using IgBLAST (via the changeo IgBLAST function v4.4.0),^55^ clustered using SCOPer (v1.3.0),^56^ germline-annotated using Dowser (v2.2.0),^57^ quantified in terms of somatic hypermutations using SHazaM (v1.2.0), all with default parameters. The pipeline was run with single samples (baseline and M06 separately) and either with 5,000 sequences (if available), with 10,000 sequences at baseline and a maximum of 10,000 sequences at M06, or with a matched approach where at baseline only as many sequences were analyzed as were later found at M06 for each patient with a maximum of 10,000 sequences. Sample overlap was defined as the proportion of the follow-up sample that contained nucleotide sequences already detected at baseline. A B-cell clonotype was defined by the members of one phylogenetic tree, whereas a T-cell clonotype was defined by identical nucleotide sequence.^60^

#### Public single-cell RNA sequencing data sets

Figures 1A and 1B contain scRNAseq data sets from Esaulova et al. (*n* = 2 RRMS patient CSF samples),^31^ Heming et al. (*n* = 5 RRMS patient CSF samples),^32^ Ramesh et al. (*n* = 12 RRMS patient CSF samples)^30^ and Schafflick et al. (*n* = 5 RRMS patient CSF samples).^33^ Figures 1C, 1D, S1A, and S3B contain the dataset from Wei et al. (*n* = 5 RRMS patient blood/CSF sample pairs and 18 RRMS patient peripheral blood samples).^12^ Figure S1B contains a scRNAseq data set combined with CITE-seq data by 10x Genomics (PBMCs from one HD).^34^ Figures S5D and S5E contain the scRNAseq dataset from Pappalardo et al. (*n* = 5 RRMS patient and *n* = 6 HD blood/CSF sample pairs).^42^ The data from Pappalardo et al. were financially supported by the NIH and downloaded from NIH’s dbGaP (accession phs002222.v2.p1). Single-cell annotation of CSF was performed using “*aCSF*”.^29^

### Quantification and statistical analysis

P values were determined by application of linear models when comparing disease groups (Figures 4B–4D) and in Figure S4, or by linear mixed models for paired comparisons (Figures S2, 3A, 3B, 4A, S5A, and S5B). All models were adjusted for age, sex, history of fingolimod treatment (5/35 MS patients), disease subgroup (PPMS or RRMS), and in case of mixed models a random intercept for each study participant. Wilcoxon signed-rank tests were used in Figures S1A and S4D.
