# Supplementary material for: B- and T cell receptor sequencing elucidates characteristics of lymphocyte depletion by ocrelizumab
Source: iScience. 2025 Jul 7;28(8):113068. doi: 10.1016/j.isci.2025.113068 (PMC12302243; doi:10.1016/j.isci.2025.113068)
Supplement: Document S1. Figures S1–S5 [file mmc1.pdf]

iScience, Volume *28*

Supplemental information

B- and T cell receptor sequencing elucidates characteristics of lymphocyte depletion by ocrelizumab

Tilman Schneider-Hohendorf, Christian Wünsch, Andreas Schulte-Mecklenbeck, Lisa Revie, Catarina Raposo, Nicolas Strauli, Björn Tackenberg, Jan D. Lünemann, Catharina C. Gross, Luisa Klotz, Heinz Wiendl, and Nicholas Schwab

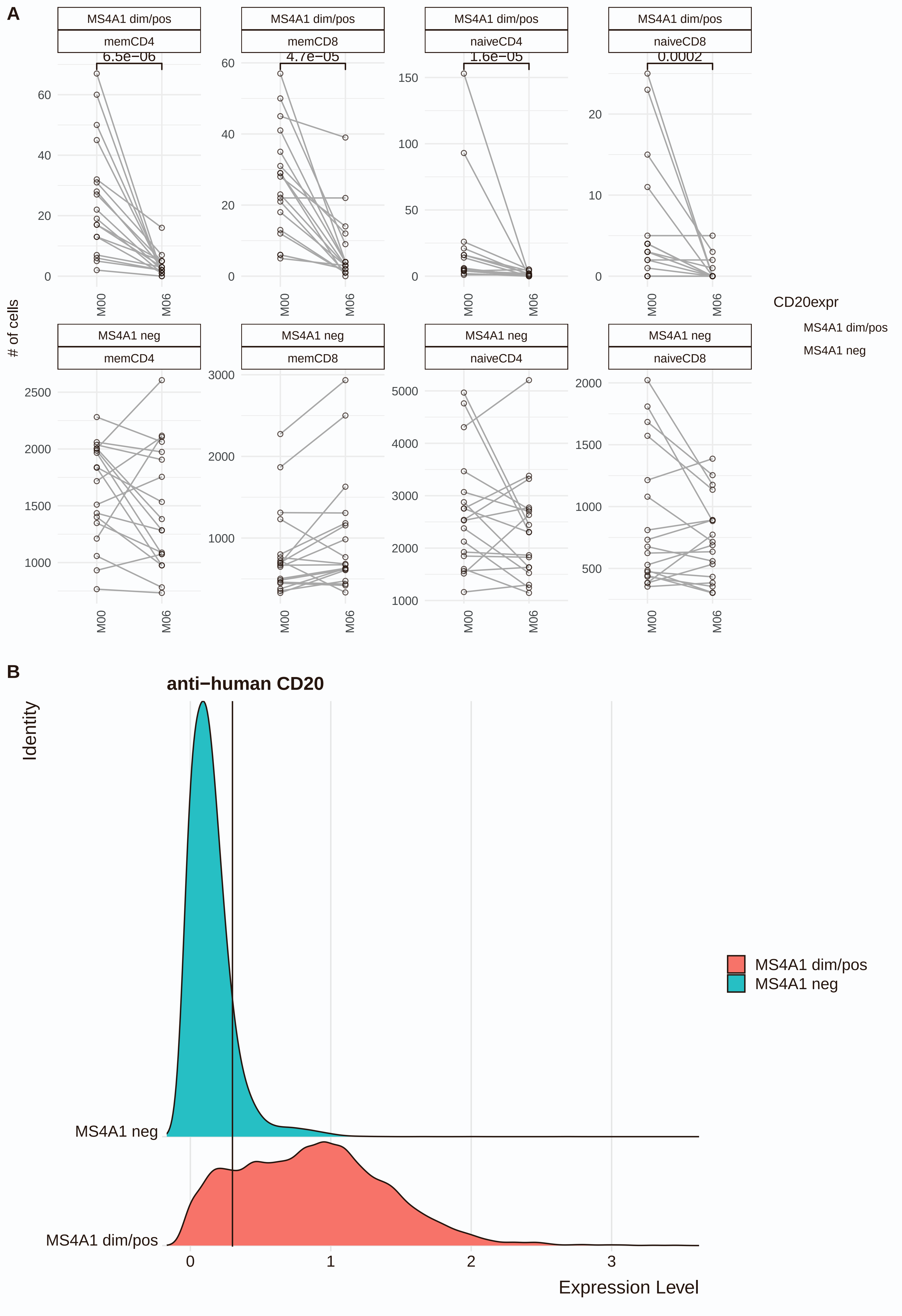
A
MS4A1 dim/pos
memCD4
6.5e−06
memCD8
4.7e−05
naiveCD4
1.6e−05
naiveCD8
0.0002
MS4A1 neg
# of cells
M00
M06
CD20expr
MS4A1 dim/pos
MS4A1 neg
B
anti−human CD20
Identity
MS4A1 neg
MS4A1 dim/pos
Expression Level
MS4A1 dim/pos
MS4A1 neg

Fig. S1: *MS4A1*-dependent depletion of CD4+ and CD8+ T cells in peripheral blood by ocrelizumab treatment and *MS4A1* RNA expression in relation to CD20 protein expression

A: The plots depict a previously published scRNAseq data set of PBMC from RRMS patients before (M00) and six months alongside ocrelizumab treatment (M06) [12]. The upper panels show CD4+ and CD8+ memory (memCD4 and memCD8) and naïve (naiveCD4 and naiveCD8) T-cell numbers with *MS4A1* expression > 0 (MS4A1dim/pos); The lower panels show CD4+ and CD8+ memory (memCD4 and memCD8) and naïve (naiveCD4 and naiveCD8) T-cell numbers without *MS4A1* expression (MS4A1 neg). B: 10x Genomics data set[34] of 19,296 rested PBMC of one healthy individual subjected to scRNAseq and stained with 148 flow cytometry antibodies (TotalSeq™-C Human Universal Cocktail, v1.0 (BioLegend, Cat#G900004) including anti-CD20. Upper panel depicts cells classified as MS4A1-negative cells (MS4A1 transcripts<1) according to Fig. 1, right panel depicts cells classified as MS4A1-positive cells (MS4A1 transcripts≥1) according to Fig. 1.

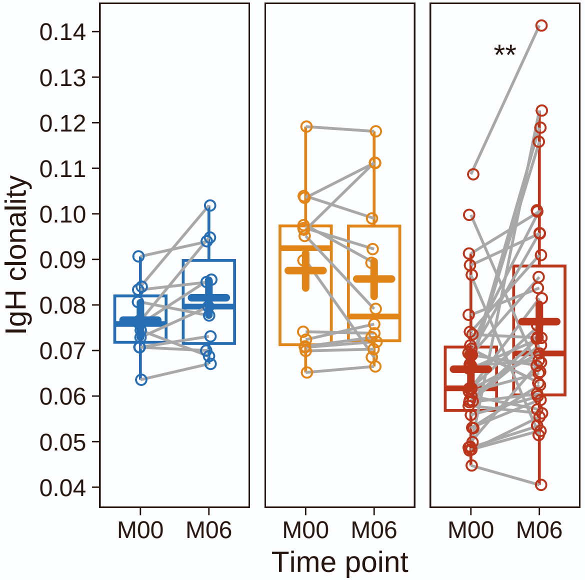

IgH clonality
0.14
0.13
0.12
0.11
0.10
0.09
0.08
0.07
0.06
0.05
0.04
**
M00
M06
M00
M06
M00
M06
Time point

Fig. S2 IgH clonality increases after ocrelizumab-associated B-cell depletion

IgH clonality, defined by frequency distribution of IgH clonotypes, where a IgH clonotype is defined by IgH sequences grouped into one phylogenetic tree; Shown are healthy controls (HD) before the first (M00) and after the second SARS-CoV-2 mRNA vaccination (M06) (blue dots and boxes), MS patients during long-term natalizumab therapy (NAT) (M00 and M06 six months apart) (orange dots and boxes) and MS patients before (M00) and six months after the start of ocrelizumab therapy (OCR) (M06) (red dots and boxes). ** indicates a p value < 0.01. Boxes indicate the 25% and 75% percentile and median, whiskers indicate 1.5x inter-quartile range, + indicates the mean.

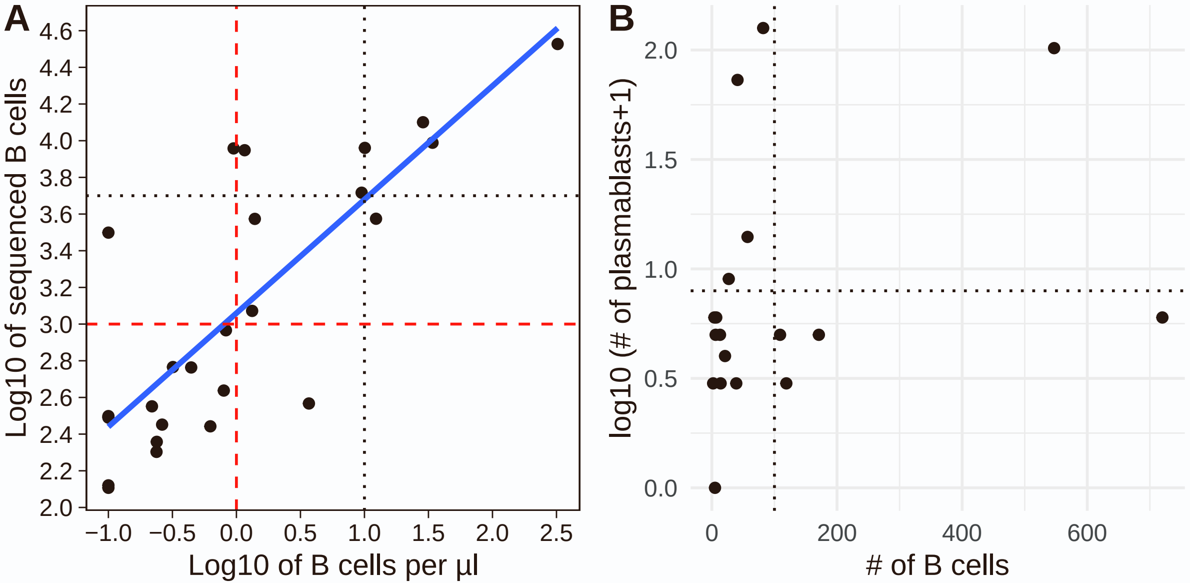
A
B
Log10 of sequenced B cells
Log10 of B cells per µl
4.6
4.4
4.2
4.0
3.8
3.6
3.4
3.2
3.0
2.8
2.6
2.4
2.2
2.0
−1.0
−0.5
0.0
0.5
1.0
1.5
2.0
2.5
log10 (# of plasmablasts+1)
# of B cells
2.0
1.5
1.0
0.5
0.0
0
200
400
600

Figure S3: Correlation of flow cytometry-derived and sequencing-derived B-cell counts and validation of bimodal B-cell distribution after initial depletion in an independent data set

A: The plot correlates the number of B cells quantified by flow cytometry (X-axis) to the number of sequenced B cells (Y-axis), both six months after the start of ocrelizumab therapy. The blue line indicates a linear regression. The black dotted lines indicate the flow cytometry-based cutoff of 10 B cells per µl corresponding to 5000 sequenced B cells. The red dashed lines indicate a IgH sequencing-based cutoff at 1000 B cells, corresponding to 1 B cell per µl. B: scRNAseq data from blood of 18 MS patients six months after the initial ocrelizumab treatment cycle, derived from a previously published study using the original cluster annotation for “B cells” and “plasmablasts”[12]. X-axis shows number of B cells, Y-axis shows log10 of number of plasmablasts + 1).

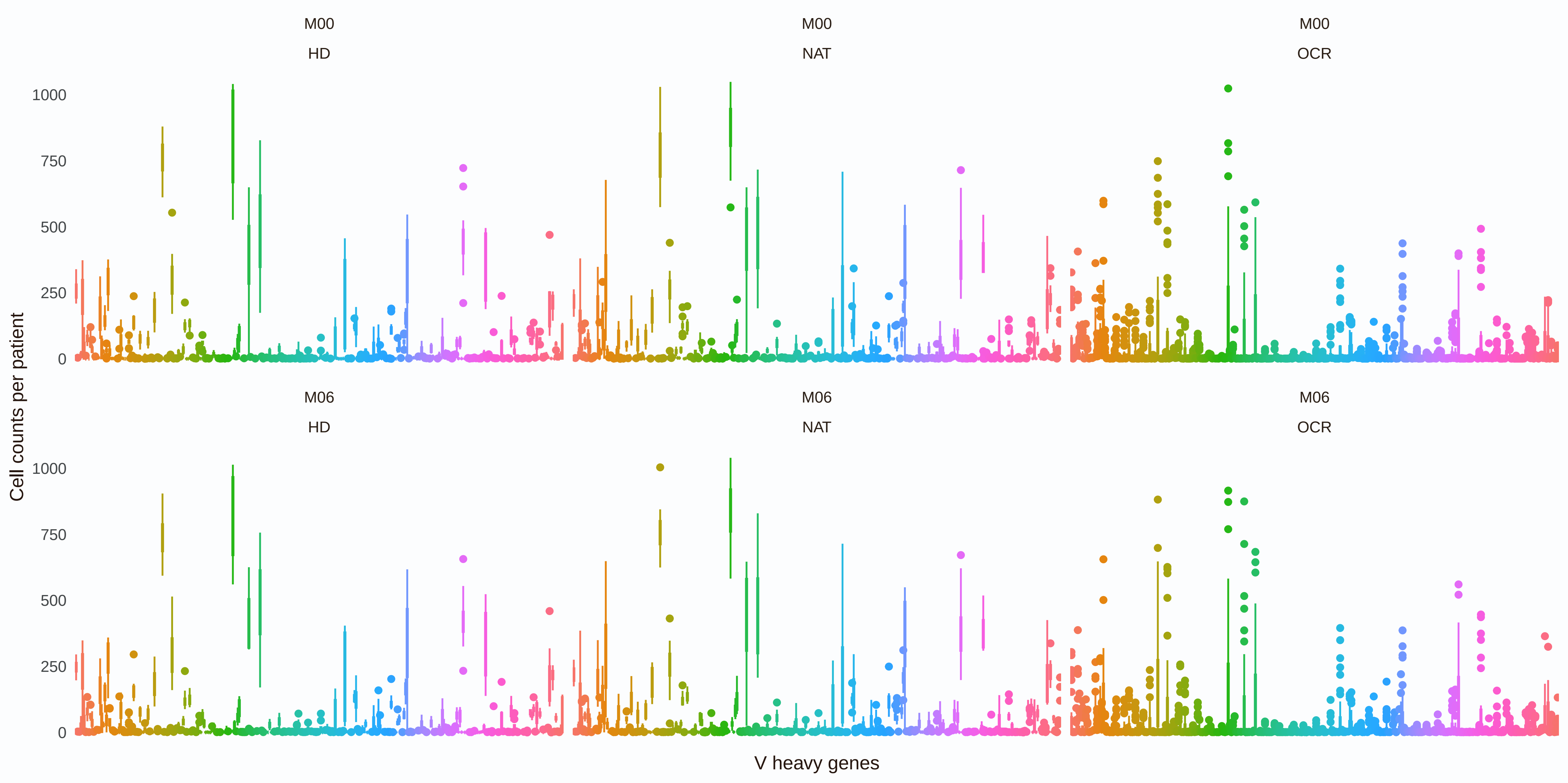

M00
HD
M00
NAT
M00
OCR
M06
HD
M06
NAT
M06
OCR
1000
750
500
250
0
Cell counts per patient
V heavy genes

Figure S4: IgH variable heavy chain gene usage before compared to six months after the start of ocrelizumab therapy

IgH variable heavy chain usage before (M00, upper right panel) and after the start of ocrelizumab therapy (M06, lower right panel), as well as in NAT (middle panels) and HD controls (left panels). Boxes indicate the 25% and 75% percentile and median, whiskers indicate 1.5x inter-quartile range.

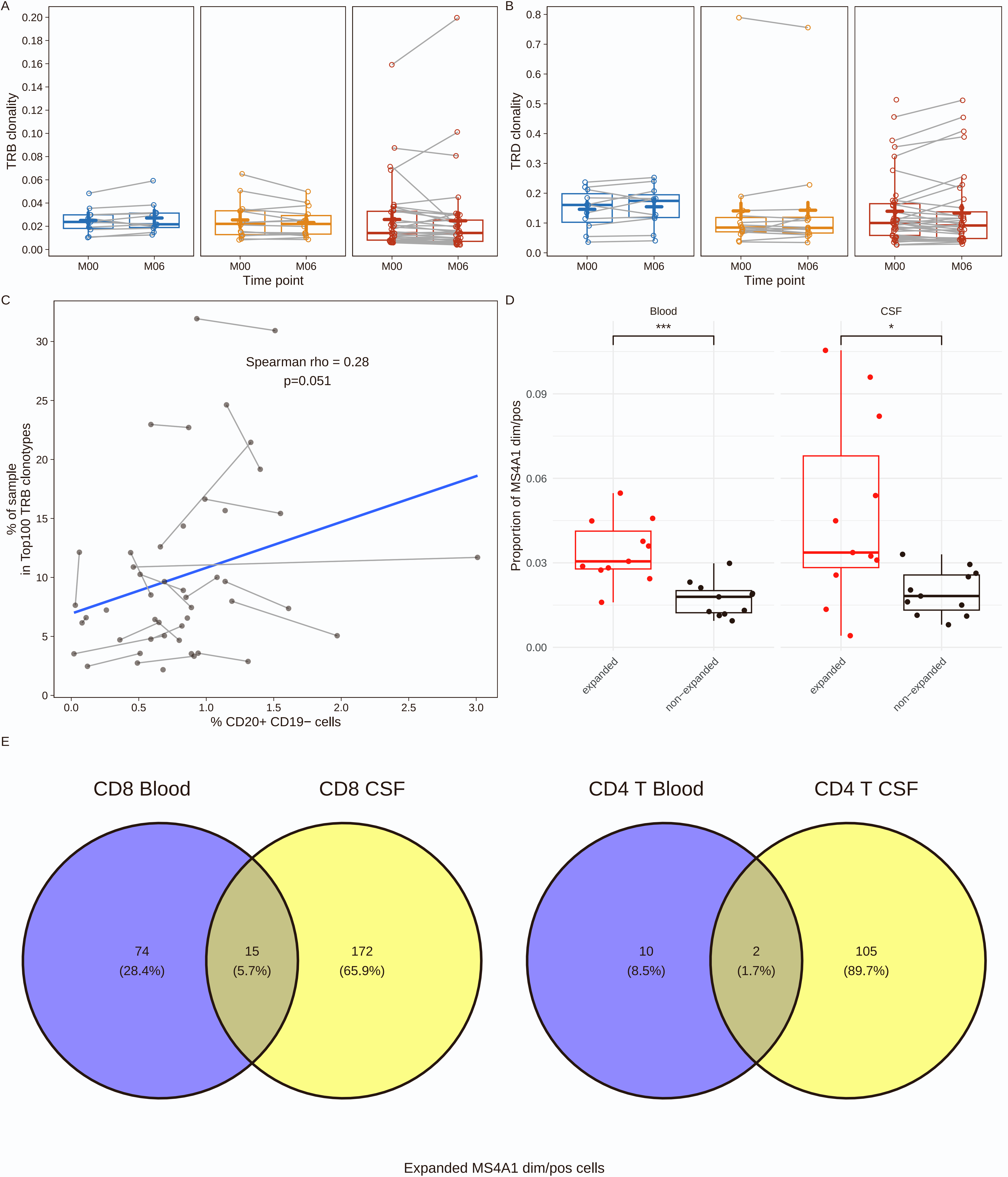
A
TRB clonality
0.20
0.18
0.16
0.14
0.12
0.10
0.08
0.06
0.04
0.02
0.00
M00
M06
Time point
B
TRD clonality
0.8
0.7
0.6
0.5
0.4
0.3
0.2
0.1
0.0
C
% of sample
in Top100 TRB clonotypes
Spearman rho = 0.28
p=0.051
% CD20+ CD19− cells
0.0
0.5
1.0
1.5
2.0
2.5
3.0
30
25
20
15
10
5
0
D
Blood
CSF
***
*
Proportion of MS4A1 dim/pos
0.09
0.06
0.03
0.00
expanded
non−expanded
E
CD8 Blood
CD8 CSF
74
(28.4%)
15
(5.7%)
172
(65.9%)
CD4 T Blood
CD4 T CSF
10
(8.5%)
2
(1.7%)
105
(89.7%)
Expanded MS4A1 dim/pos cells

Figure S5: TRB and TRD clonality are not modified by ocrelizumab therapy and clonally expanded CD20dim T cells are traceable from blood to CSF

The plots depict clonality of TRB (A) and TRD (B) repertoires of the OCR, NAT and HD cohorts at baseline (M00) and six-month follow-up (M06). C depicts a correlation between % CD20+ CD19- cells and % of TRB sample occupied by the top 100 clonotypes combined at baseline and at six-month follow-up. Lines connect the two samples from the same patient, the blue line indicates a linear regression, and given are the Spearman rho value of 0.28 and the corresponding p value of 0.051. D depicts blood (left panel) and CSF (right panel) immune cells with either expanded or non-expanded TRB sequences. E depicts the TRB sequence overlap determined by amino acid and variable beta chain gene matching of expanded *MS4A1*-positive cells from blood (blue circle) and CSF (yellow circle). D and E contain scRNAseq of n=5 MS patient blood/CSF pairs and n=6 HD blood/CSF pairs from a previously published data set[52]. Boxes indicate the 25% and 75% percentile and median, whiskers indicate 1.5x inter-quartile range, + indicates the mean.
